# Community characteristics and relationship between gut microbiota and intratumoral microbiota in hepatocellular carcinoma

**DOI:** 10.3389/fimmu.2024.1500863

**Published:** 2025-01-10

**Authors:** Huangpeng Lin, Zexian Ma, Jin Li, Heping Zhu, Xuefeng Huang, Huimin Chen, Liang Tu, Yifan Lian, Yongjie Su

**Affiliations:** ^1^ The School of Clinical Medicine, Fujian Medical University, Fuzhou, China; ^2^ Department of Health Management Center, Xiang’an Hospital of Xiamen University, School of Medicine, Xiamen University, Xiamen, China; ^3^ Department of Hepatobiliary and Pancreatic Surgery, Xiamen Traditional Chinese Medicine Hospital, Xiamen, China; ^4^ Department of Hepatobiliary and Pancreatic Surgery, Zhongshan Hospital of Xiamen University, School of Medicine, Xiamen University, Xiamen, China; ^5^ Department of Gastroenterology, The National Key Clinical Specialty, Zhongshan Hospital of Xiamen University, School of Medicine, Xiamen University, Xiamen, China

**Keywords:** hepatocellular carcinoma, transarterial chemoembolization, hepatic artery infusion chemotherapy, programmed cell death protein-1 inhibitors, lenvatinib, gut microbiota, intratumoral microbiota

## Abstract

**Background:**

The combination of local therapy with lenvatinib and programmed cell death protein-1 (PD-1) inhibitors represents an emerging treatment paradigm for unresectable hepatocellular carcinoma (uHCC). Our study sought to investigate the interrelationship between gut microbiota and intratumoral microbiota in the context of triple therapy, with a view to identifying potential biological markers.

**Methods:**

The gut microbial community profiles of patients with primary untreated hepatocellular carcinoma (HCC) and those treated with local therapy combined with lenvatinib and PD-1 inhibitors were analyzed by 16S rRNA gene amplicon sequencing. Additionally, microbial community profiles of tumor tissues of patients with HCC and normal liver tissues were analyzed.

**Results:**

In our investigation, we observed that patients with HCC who received triple therapy exhibited a notable enhancement in the abundance of Actinobacteriota and a considerable decrease in *Escherichia Shigella*. Patients who received hepatic artery infusion chemotherapy (HAIC) in combination with levatinib and PD-1 inhibitors exhibited significantly elevated levels of *Faecalibacterium prausnitzii* and *Bacteroides stercoris* in comparison to those who received transarterial chemoembolization (TACE) in combination with levatinib and PD-1 inhibitors. Furthermore, a notable decline in microbial diversity was observed within HCC tumors in comparison to normal liver tissues. The gut and intratumoral microbiota in HCC patients exhibited a high degree of similarity to the microbes present at the phylum level.

**Conclusions:**

Gut microbiota is connected to triple therapy with local therapy combined with lenvatinib and PD-1 inhibitors for HCC. These discoveries underscore the potential of utilizing gut microbiota and intratumoral microbiota as biomarkers, as well as the possibility of triple therapy in the management of HCC.

## 1 Introduction

Primary liver cancer represents the sixth most common tumor worldwide and the third chief causal factor in cancer-related mortalities. This has a profound impact on the lives and health of patients (1). Hepatocellular carcinoma (HCC) represents the most prevalent form of primary liver cancer, accounting for approximately 90% of cases. Surgery remains the most important treatment for HCC (2). However, HCC is often found to be in an intermediate to advanced stage and cannot be eradicated by surgical resection (3). In recent years, tyrosine kinase inhibitors (TKIs), immune checkpoint inhibitors, and localized therapies have emerged as new weapons in the fight against HCC. The combined application of these treatments has also demonstrated promising results in clinical settings (4). Translational therapy for HCC refers to downstaging unresectable hepatocellular carcinoma (uHCC) through various therapeutic approaches, transforming it into surgically resectable HCC or achieving long-term survival (5). The triple therapy based on TKIs and PD-1 inhibitors combined with local therapy has brought the treatment of uHCC into a new era (6). Lenvatinib is the first-line TKIs for the treatment of uHCC, and several studies have demonstrated that local therapy combined with lenvatinib and PD-1 inhibitors for uHCC has better benefits and broader prospects (7–11).

The human gastrointestinal tract contains over 100 trillion microorganisms, such as bacteria, fungi, viruses, and archaea. These microorganisms play a vital role in various physiological activities like digestion, metabolism, and immunity (12). Recent research has revealed that gut microbiota also have a substantial impact on the treatment and prognosis of tumors. It is anticipated that they will serve as intervening factors and novel biomarkers for tumor therapy, which is of significant importance for the prompt diagnosis and tailored management of tumors in clinical settings (13). Microorganisms are found in tumor tissues. With the advancement of second-generation sequencing technology, numerous studies have confirmed the presence of microorganisms in various types of carcinomas such as hepatocellular, pancreatic, gastric, colorectal, esophageal, lung, prostate, renal cell, neurogliomas, melanomas, squamous cell carcinomas of the oral cavity, and ovarian and cervical carcinomas (14). The tumor microenvironment (TME) includes non-cancerous cells and constituents within tumors, such as immune cells, endothelial cells, and cytokines. The communications between tumor cells and TME are crucial in the progression of tumors and the response to therapeutic interventions. Targeting TME therapy presents a new opportunity and challenge (15). In recent years, the intratumoral microbiota has been identified as a constituent element component of TME (16). Intratumoral microbiota is closely linked to tumorigenesis and progression, potentially contributing to cancer development and progression by inducing DNA mutations, activating oncogenic pathways, promoting chronic inflammation, initiating the complement system, and mediating metastasis (17). Additionally, intratumoral microbiota is associated with anti-tumor immune response. There are differences in the species abundance of intratumoral microbiota between responders and non-responders to PD-1 inhibitors (18).

Previous research has shown that the gut microorganisms of patients with HCC differ tremendously from those of healthy individuals. This indicates that the composition of gut microbiota can potentially serve as a biological marker (19). Additionally, the presence of microorganisms in tumors can be used as a biological indicator to predict surgical outcomes and prognosis (20). Microorganisms and their byproducts have an influence on the gut-liver axis, which facilitates bi-directional interactions between the gastrointestinal and hepatic systems (21). To further investigate this, stool specimens were collected from 26 patients with previously untreated HCC and 26 HCC patients after triple therapy, as well as tumor tissues from 19 tumor tissues and 15 normal liver tissues. The microbiological communities were subjected to analysis through the utilization of 16S rRNA gene amplicon sequencing to identify differences in gut microbiota between patients with HCC after triple therapy and those with previously untreated HCC. This was done in order to identify differential flora, to explore the correlation and differences between gut microbiota and intratumoral microbiota in HCC patients, and to identify target microbes that can diagnose early and predict therapeutic efficacy.

## 2 Materials and methods

### 2.1 Patient enrollment and sample collection

We conducted a study from September 2023 to June 2024 at Zhongshan Hospital of Xiamen University involving 26 patients diagnosed with untreated HCC, 16 patients with HCC treated with local therapy combined with lenvatinib and PD-1 inhibitors, and 10 patients treated at Xiamen Hospital of Traditional Chinese Medicine with the same therapy. The previously untreated HCC patients were referred to as group U. They were enrolled based on the criteria of having untreated HCC before admission and a clinical diagnosis of HCC according to the Chinese Primary Liver Cancer Diagnosis and Treatment Standard 2022 edition. The patients treated with local therapy combined with lenvatinib and PD-1 inhibitors were referred to as group T. They were enrolled based on the criteria of being over 18 years old, having an PS score of 0-2, having a clinical diagnosis of HCC according to the Chinese Primary Liver Cancer Diagnosis and Treatment Standard 2022 edition, having a liver function grade of Child-Pugh class A or B, and not having received any previous antitumor treatment. The exclusion criteria included:1. Individuals who had taken probiotics or microbial preparations within one month of the commencement of treatment 2. Individuals who had undergone surgical procedures affecting the gastrointestinal, liver, gallbladder, or other related organs within the previous month.3. A history of other malignant neoplasms, either before or concurrent with the current condition. The local therapy included transcatheter arterial chemoembolization (TACE) and hepatic arterial infusion chemotherapy (HAIC). Patients with HCC treated with local therapy combined with lenvatinib and PD-1 inhibitors were divided into the TACE group and HAIC group, with 9 cases in the TACE group and 16 cases in the HAIC group. The patients in the U group were provided with fresh fecal samples before receiving treatment. These samples were collected in MGIEasy fecal sample collection tubes provided by MGI Tech Co., Ltd. The samples were quickly transferred to a -80°C refrigerator for preservation. Meanwhile, the patients in the T group underwent at least one period of local therapy combined with lenvatinib and PD-1 inhibitors. Fresh feces were collected from these patients when they returned to the hospital for treatment, using MGIEasy fecal sample collection tubes, and quickly transferred to a -80°C refrigerator for storage. Surgical specimens of tumor tissue from 19 patients with clinically and postoperatively confirmed HCC, which we refer to as Group I, as well as surgical specimens of normal liver tissue from 15 patients with clinically and postoperatively, confirmed benign disease (hepatic hemangioma, focal nodular hyperplasia of the liver), which we refer to as Group L, were collected from Zhongshan Hospital of Xiamen University. The tumor tissue and normal liver tissue samples were collected aseptically in the operating room, all were collected within 30 min of specimen removal, and the specimens were stored in liquid nitrogen immediately after collection and transferred to a -80°C refrigerator at the end of the procedure. Prior to enrollment, each participant signed a written informed consent form. This experiment has been reviewed by the China Clinical Trials Registry (ChiCTR2400087954).

### 2.2 DNA extraction and amplicon library preparation (DNBSEQ)

The extraction of DNA from samples was performed using the MagPure Stool DNA KF Kit B (MAGEN, Guangzhou, China), in adherence to the provided instructions. The library was prepared by 2 × Phanta Max Master Mix (VAZYME, China) polymerase, and the V3V4 variable region of 16S rDNA of bacteria was amplified by forward and reverse PCR degenerate primers F and R (338F: ACTCCTACGGGAGGCAGCAG, 806R: GGACTACHVGGGTWTCTAAT). After passing the quality control, the libraries were combined to create a DNA nanoball (DNB) containing multiple copies. These DNBs were then placed into the mesh pores on the chip using high-density DNA nano-chip technology. Subsequently, sequencing was performed on the DNBSEQ-G400 sequencing platform (BGI, Shenzhen, China) using co-probe anchored polymerization technology (cPAS).

### 2.3 Microbiological data analysis

The raw sequencing data underwent processing to obtain clean data. The sequence splicing was performed using the software FLASH (Fast Length Adjustment of Short reads, v1.2.11). To get the tags of the highly variable range, FLASH assembled pairs of reads from double-ended sequencing into a single sequence using the superimposed relation. The Amplicon Sequence Variants (ASVs) were denoised using the DADA2 (Divisive Amplicon Denoising Algorithm) method in QIIME2. This denoising method helped obtain ASVs, which are sequences with 100% similarity. Following this, the feature table was generated. After receiving the ASV representative sequences, the RDP classifier software was used to compare them with the Silva database for species annotation.

### 2.4 Statistics

Statistical analysis was conducted using SPSS version 26.0 (IBM, Armonk, NY, USA) to determine the significance of differences between groups. The Wilcoxon Rank-Sum Test was employed to compare the α-diversity of the two groups. LEfSe analysis was used to discover the biomarkers of the two groups by the nonparametric factorial Kruskal-Wallis rank-sum test. Biomarker was obtained by Phylogenetic Investigation of Communities by Reconstruction of Unobserved States(PICRUST2) (https://github.com/picrust/picrust2) Abundance prediction of KEGG functions of bacterial communities. After the prediction of the functions obtained for all the samples, the Wilcox test was used to find the differential functions between the groups and graphically presented.

## 3 Results

### 3.1 Patient information

The study included 26 individuals with a median age of 59 in the U cohort, 26 individuals with a median age of 56.5 in the T cohort, 15 individuals with a median age of 37 in the L cohort, and 19 individuals with a median age of 58 in the I cohort. No measurable differences were found for gender, age, hepatitis B, cirrhosis, BMI, history of alcohol consumption, alpha-fetoprotein (AFP), prothrombin time (PT), albumin (Alb), total bilirubin (TBil), alanine transaminase (ALT), aspartate transaminase (AST), and maximum diameter of the tumors. However, AFP, ALT, AST, and TBil were lower in group T compared to group U. Gender, age, hepatitis B, and cirrhosis were similar between groups L and I. Furthermore, no impressive differences were observed in other indicators including BMI, alcohol consumption history, AFP, PT, Alb, TBil, Alb,ALT, AST, and maximum diameter of tumors. Baseline demographics and clinical characteristics of the two groups are presented in **Tables 1**, **2**.

**Table 1:** Characteristics of previously untreated HCC patients and triple therapy for HCC patients.

Characteristic	Group T (n=26)	Group U (n=26)	P value
Sex (male/female)	26/0	26/0	NaN
Age (years)	56.5 (51.0-59.3)	59.0 (52.0-69.0)	0.309
BMI (Kg/m 2)	22.5 (21.0-24.1)	22.7 (20.9-24.5)	0.875
Hepatitis B (Yes/No)	21/5	25/1	0.082
Cirrhosis (Yes/No)	9/17	10/16	0.773
Alcohol history (yes/no)	3/23	8/18	0.089
AFP (ng/ml)	11.2 (4.6-1076.9)	72.7 (6.4-1639.3)	0.687
PT (sec)	11.3 (9.8-12.3)	10.6 (9.7-12.0)	0.239
Alb (g/L)	38.0 (34.3-40.7)	39.1 (32.4-42.4)	0.908
TBil (umol/L)	13.0 (11.3-24.2)	21.5 (14.8-27.0)	0.865
ALT (U/L)	32.2 (22.8-64.7)	40.3 (23.6-76.6)	0.446
AST (U/L)	48.2 (32.0-66.0)	76.2 (34.4-120.0)	0.701
Maximum tumor diameter (cm)	8.4 (3.9-11.5)	9.1 (4.3-13.0)	0.575

**Table 2:** Characteristics of HCC patients and benign disease patients.

Characteristic	Group L (n=15)	Group I (n=19)	P value
Sex (male/female)	8/7	18/1	0.015
Age (years)	37 (31-46.5)	58 (50.5-64)	0.001
BMI (Kg/m 2)	21.97 (21.15-26.04)	23.88 (22.87-25.32)	0.659
Hepatitis B (Yes/No)	1/14	17/2	0.001
Cirrhosis (Yes/No)	0/15	12/7	0.001
Alcohol history (yes/no)	2/13	6/13	0.213
AFP (ng/ml)	2.5 (2.15-2.9)	14 (3.55-610.6)	0.059
PT (sec)	9.63 (8.88-10.4)	10.1 (9.33-11)	0.172
Alb (g/L)	43.9 (42.7-47.7)	44.7 (38.45-45.8)	0.082
TBil (umol/L)	12.7 (11.4-14.7)	14.4 (11.95-20.35)	0.893
ALT (U/L)	24.2 (16.45–27.15)	30.4 (26.5-52.05)	0.979
AST (U/L)	21.5 (18.5-24.9)	33.8 (27.65-53.3)	0.569

### 3.2 Microbial diversity and composition of Group T and Group U

**Figure 1:**
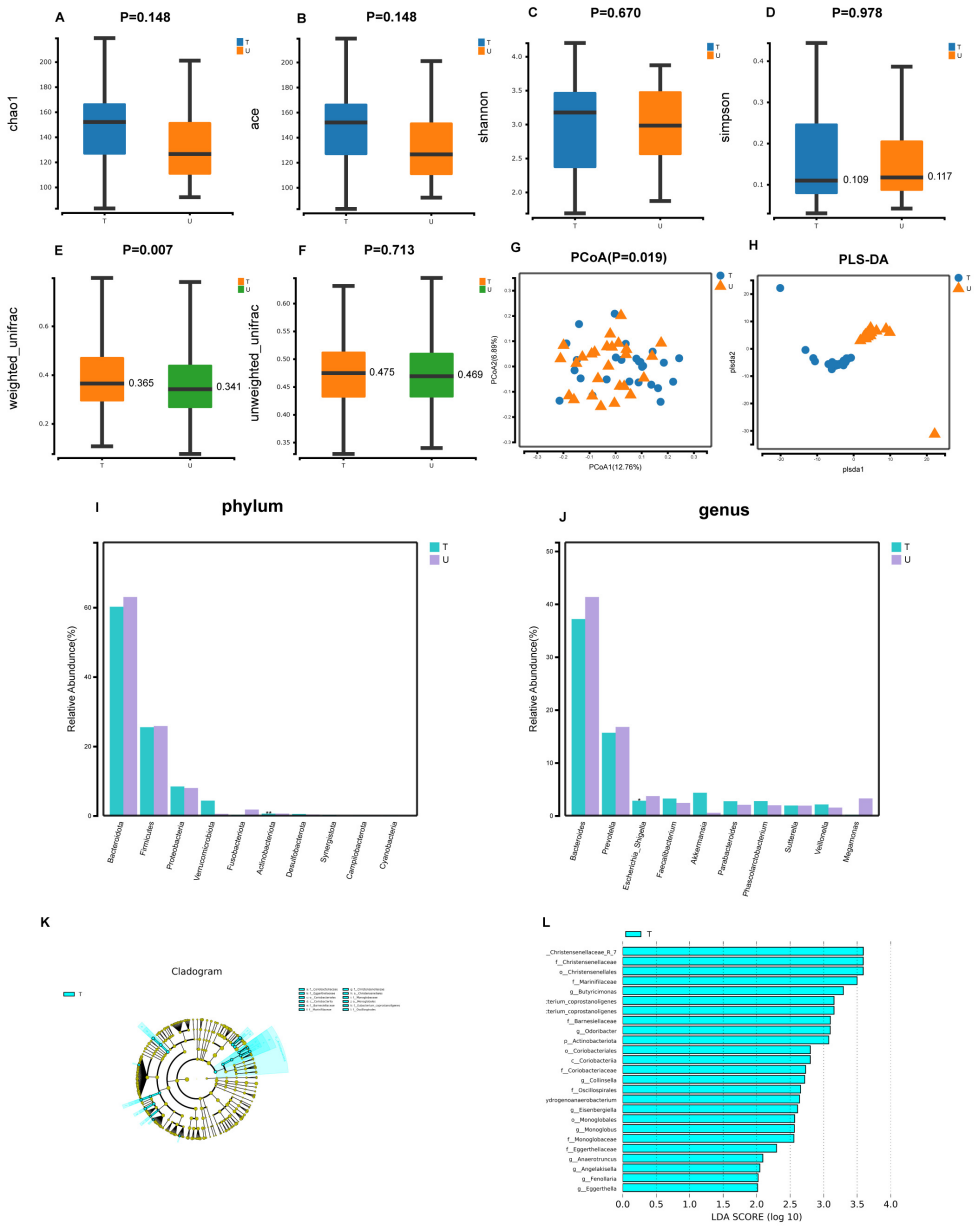
Gut microbial diversity and taxonomic composition in patients with primary untreated HCC patients with HCC treated with local therapy combined with lenvatinib and PD-1 inhibitors. **(A-D)** Between-group α-diversity indices from **(A–D)** are chao1, ace, shannon, simpson. **(E, F)** β-diversity differences between groups based on weighted and unweighted unifrac distance. **(G)** Between-group PCoA analysis based on Unweighted unifrac distance. **(H)** Between-group PLS-DA analysis i.e. partial least squares discriminant analysis. **(I, J)** Histograms of gut microbial species at the phylum and genus level, marked with "***" if P < 0.001, "**" if 0.001 <= P <= 0.01, "**" marker; if 0.01 < P <= 0.05 "*" marker, if P > 0.05 no marker. **(K)** LEfSe clustering plot of two groups of gut microbiota. **(L)** LEfSe LDA plot of two groups of gut microbiota (LDA score [log10]>2).

In our study using 16S rRNA gene amplicon sequencing, we discovered higher intestinal microbial diversity indices (Chao1, Ace, and Shannon) in group T compared to group U. Additionally, the Simpson index was lower in group T. This indicates that the α-diversity of intestinal microorganisms was increased in patients with HCC after receiving triple therapy of local therapy combined with lenvatinib and PD-1 inhibitors, compared to patients with primary untreated HCC. Nevertheless, this discrepancy was not found to be sufficiently statistically. (P>0.05) (**Figures 1A–D**). Furthermore, we identified notable discrepancies in the statistical significance of β-diversity based on weighted unifrac distance between groups T and U, as shown by P < 0.05 for Principal Co-ordinates Analysis(PCoA) and dispersion between samples of the two groups in Partial Least Squares Discriminant Analysis(PLS-DA) plots, indicating the microbial distinction between the two groups (**Figures 1E–H**). Furthermore, our statistical analyses at the phylum levels and genus levels exhibited differences in the main microbial constitutions between the two groups. At the phylum level, Bacteroidota, Firmicutes, and Proteobacteria had the highest abundance in group T, while Actinobacteriota had a lower abundance, and this difference was significant (P<0.01) (**Figure 1I**). At the genus level, *Bacteroides*, *Prevotella*, and *Escherichia Shigella* had the highest abundance in group T, while *Escherichia Shigella* had a lower abundance, and this difference was significant (P<0.05) (**Figure 1J**). Additionally, the results of the Lefse analysis highlighted significant differences in the fecal microbial composition between the two groups. Group T showed a significant abundance of *Christensenellaceae R-7*, *Christensenellaceae*, *Christensenellales*, *Marinifilaceae*, *ButyrICIsmonas*, *Eubacterium coprostanoligenes*, *Barnesiellaceae,Odoribacter*, *Actinobacteriota* (LDA score [log10]>4) (**Figures 1K, L**).

### 3.3 Microbial diversity and composition of TACE and HAIC groups

**Figure 2:**
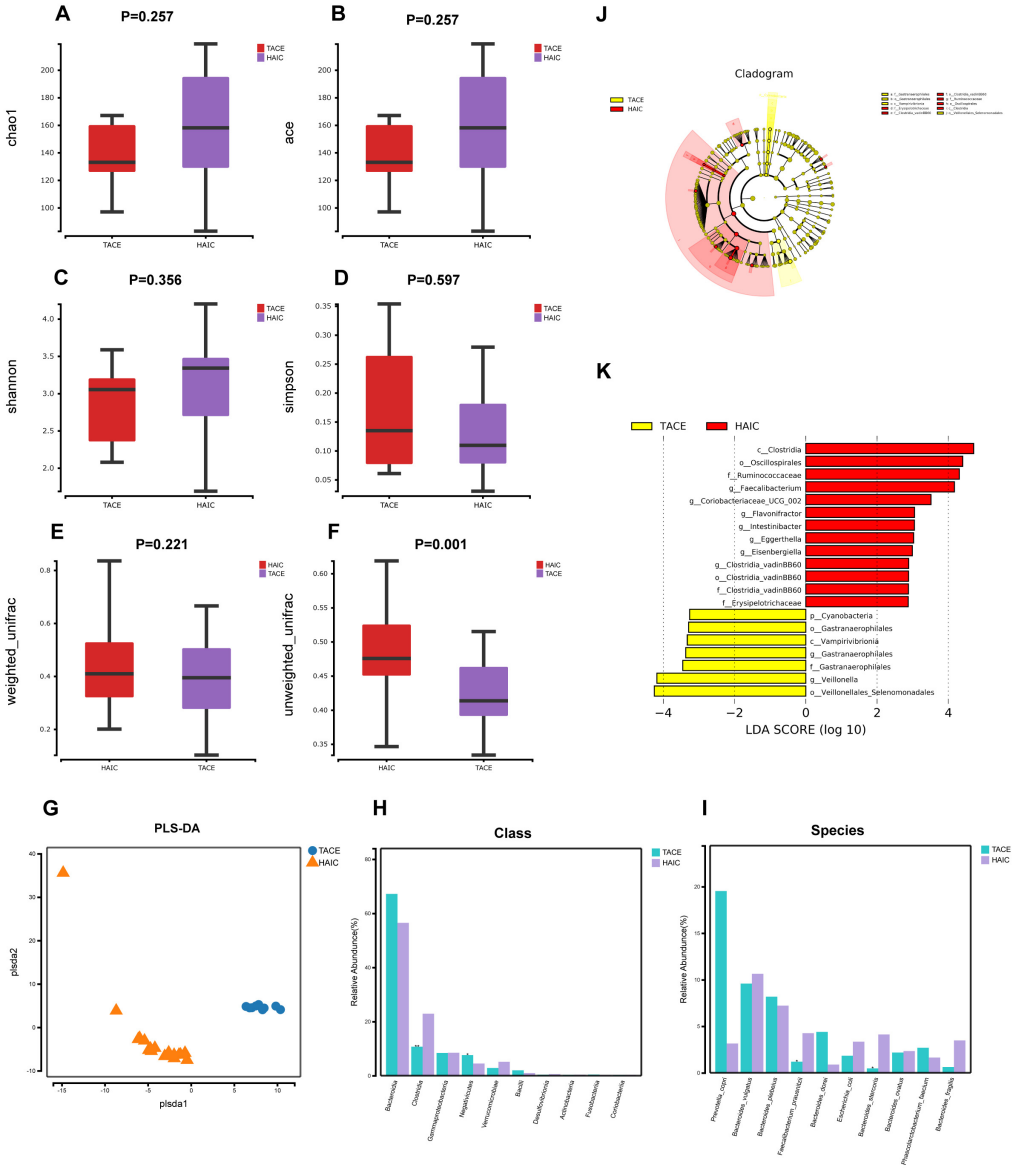
Gut microbial diversity and taxonomic composition in HCC patients with TACE combined with lenvatinib and PD-1 inhibitors and HCC patients with HAIC combined with lenvatinib and ICIs. **(A-D)** Between-group α-diversity indices from **(A–D)** are chao1, ace, shannon, simpson. **(E, F)** β-diversity differences between groups based on weighted and unweighted unifrac distance. **(G)** β-diversity differences between groups by PLS-DA analysis i.e., Partial Least Squares Discriminant Analysis. **(H)** Comparison of keystone species differences at the class level. **(I)** Comparative bar chart of key species differences at the species level. **(J)** LEfSe clustering plot of two groups of gut microbiota. **(K)** LEfSe LDA plot of two groups of gut microbiota (LDA score [log10]>2).

A comparative analysis of the alterations in gut microbial diversity between the TACE and HAIC cohorts revealed that the Chao1, Ace and Shannon indices exhibited higher values in the HAIC cohort compared to the TACE cohort. Conversely, the Simpson index was lower in the TACE group. This indicates that the gut microbial diversity was greater in the HAIC group, although the distinction was not statistically measurable (P>0.05) (**Figures 2A–D**). A statistically meaningful intergroup difference in β-diversity of gut microorganisms was observed between the TACE and HAIC groups (P < 0.01). Furthermore, the PLS-DA plot demonstrated a distinct separation between the two groups, indicating a notable divergence in the gut microbial composition between the two groups. (**Figures 2E–G**). Statistical analyses were performed at both the class and species levels to ascertain the predominant microorganism constitution of the two groups. At the class level, Clostridia (P<0.01) and Negativicutes (P<0.05) showed a significant difference, with higher abundance in the HAIC group (**Figure 2H**). At the species level, *Faecalibacterium prausnitzii* (P<0.05) and *Bacteroides stercoris* (P<0.01) exhibited differences, with higher abundance in the HAIC group (**Figure 2I**). Lefse analysis showed that Veillonellales Selenomonadales and *Veillonella* had significant presence in the feces of the TACE group (LDA score [log10]>4), while in the fecal microorganisms of the HAIC group, Clostridia, *Oscillospirales*, Ruminococcaceae, and *Faecalibacterium* were deemed significant (LDA score [log10]>4) (**Figures 2J, K**).

### 3.4 Microbial diversity and composition of Group I and Group L

**Figure 3:**
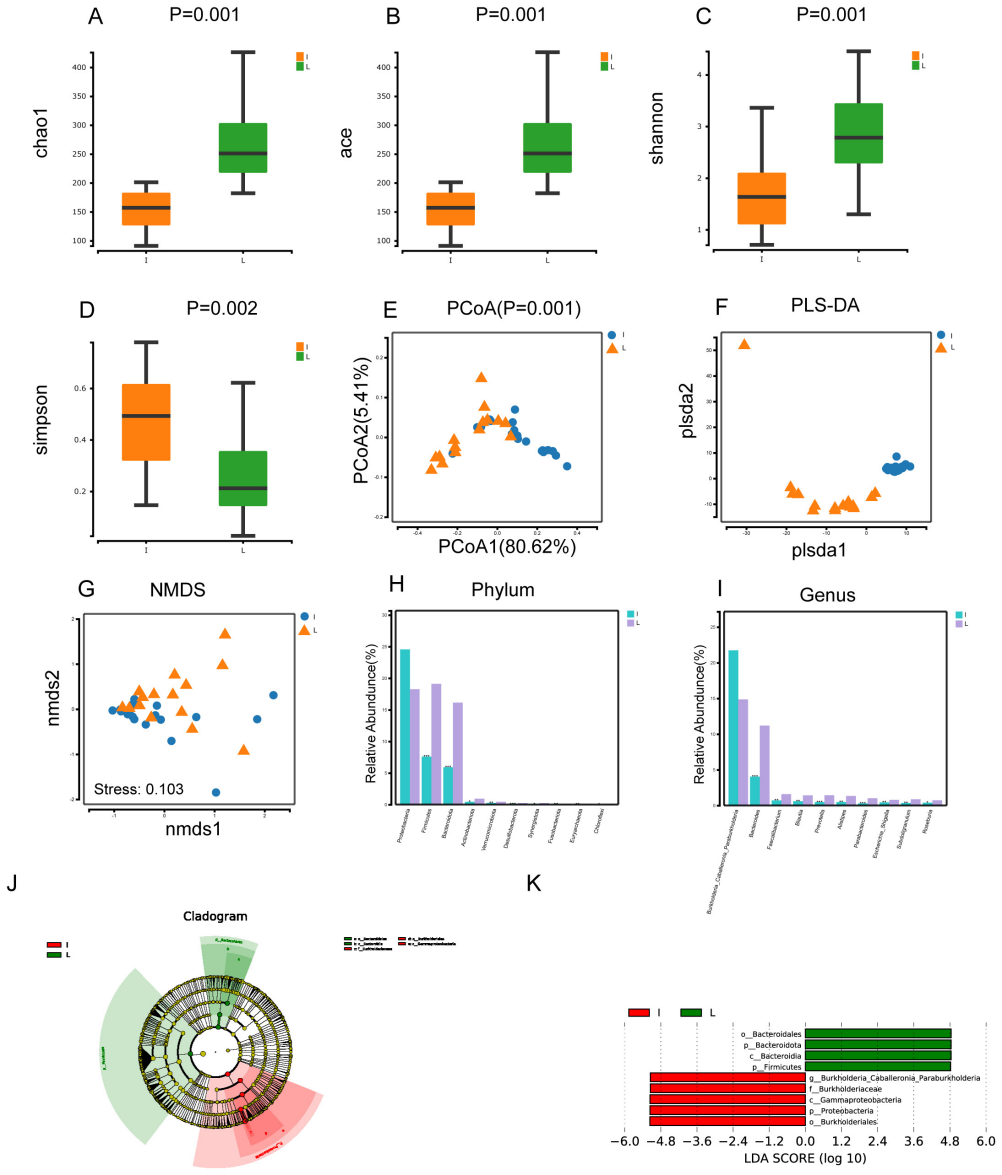
Microbial diversity and taxonomic composition of tumor tissues and normal liver tissues from HCC patients. **(A-D)** Intergroup α-diversity indices from **(A–D)** are chao1, ace, shannon, simpson. **(E)** Intergroup PCoA analysis based on weighted unifrac distance **(F)** Between-group PLS-DA analysis was partial least squares discriminant analysis **(G)** Intergroup NMDS analysis i.e. non-metric multidimensional scaling method, stress<0.2 its graphs have some interpretive significance **(H, I)** Comparative plots of microbial keystone species differences at the phylum and genus level, marked with "***" if P < 0.001 , "***" if 0.001 <= P <= 0.01, "**" marker; if 0.01 < P <= 0.05, then "*" marker; if P > 0.05 then no marker **(J)** LEfSe clustering plot of two groups of microbes **(K)** LEfSe LDA plot of two groups of microbes (LDA score [log10]>4.8).

In our study, we observed that the Chao1, Ace, and Shannon indices were superior in group L compared to group I, while the Simpson indices were lower in group L than in group I. This suggests that the α-diversity of gut microbial was greater in group L (**Figures 3A–D**). The P value was less than 0.05 for the PCoA of group I and group L, indicating that the variations between the two groups were statistically meaningful (**Figures 3E, F**). The dispersion of the samples between the two groups was also visible in the PLS-DA plot, suggesting distinct differences in the microorganisms of the two groups. Non-metric Multidimensional Scaling(NMDS) analysis is founded upon the distance matrix of the samples, demonstrating evident discrepancies in the specific distance distribution of the samples. The data of Group I were mainly distributed in the third quadrant, while the data of Group L were primarily distributed in the first and fourth quadrants. The Stress value was less than 0.200, signifying statistical significance (**Figure 3G**). Comparative genomic analysis at the phylum level revealed that the species composition of the two groups exhibited significant disparities, particularly in the prevalence of Firmicutes, Bacteroidota, and Desulfobacterota. (P< 0.001) (**Figure 3H**). Similarly, at the genus level, *Bacteroides*, *Prevotella*, and *Parabacteroides* showed highly significant differences (P< 0.001) (**Figure 3I**). Lefse analysis indicated that in group L, Bacteroidales, Bacteroidota, Bacteroidia, and Firmicutes were significant (LDA score [log10]>4.8). Additionally, the microorganisms Burkholderiales, Proteobacteria, Gammaproteobacteria, *Burkholderiaceae*, and *Burkholderia Caballeronia Paraburkholderia* also had significant (LDA score [log10]>4.8) (**Figures 3J, K**).

### 3.5 Composition of groups U and I

**Figure 4:**
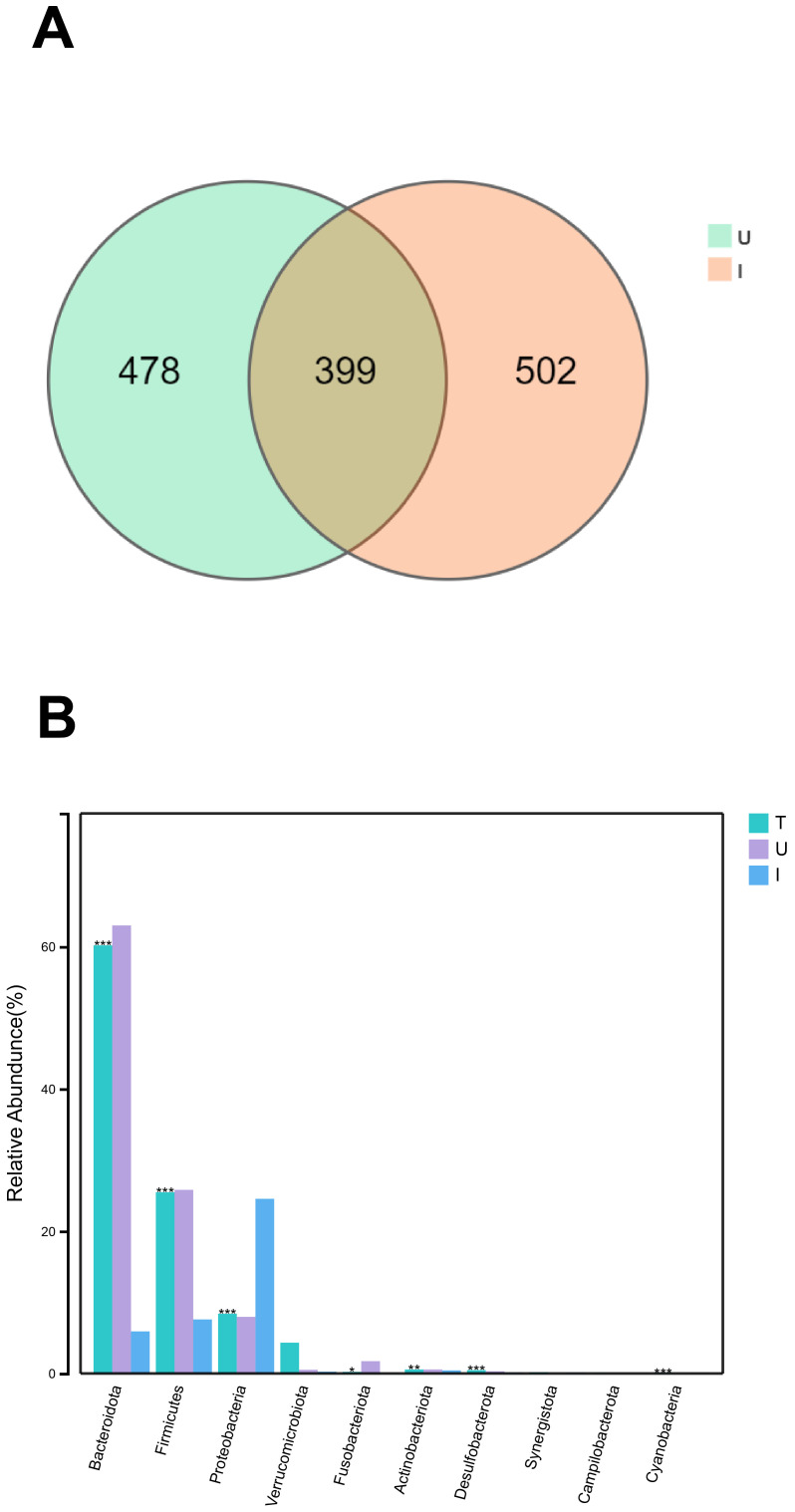
The same and different of group U and I **(A)** Venn diagram of microorganisms of group U and group I. **(B)** Histogram of species composition of group T and U and I microorganisms at the phylum level , marked with “***” if P < 0.001, “**” if 0.001 <= P <= 0.01, “** “ marker; if 0.01 < P <= 0.05, “*” marker; if P > 0.05, no marker.

In our study, we conducted a comparison of the gut microbes in HCC patients with the microbes found in the tumors of HCC patients. We then analyzed the distribution of the microbes in both groups. The Venn diagram illustrates that group U contains 877 unique microorganisms and the group I contains 901 unique microorganisms, with 399 overlapping microorganisms (**Figure 4A**). At the phylum level, we observed that the highest abundance of microorganisms Bacteroidota, Firmicutes, and Proteobacteria were found in group U, while the highest abundance of microorganisms Proteobacteria, Bacteroidota, and Firmicutes were found in group I (**Figure 4B**).

### 3.6 PICRUSt2-based functional prediction

**Figure 5:**
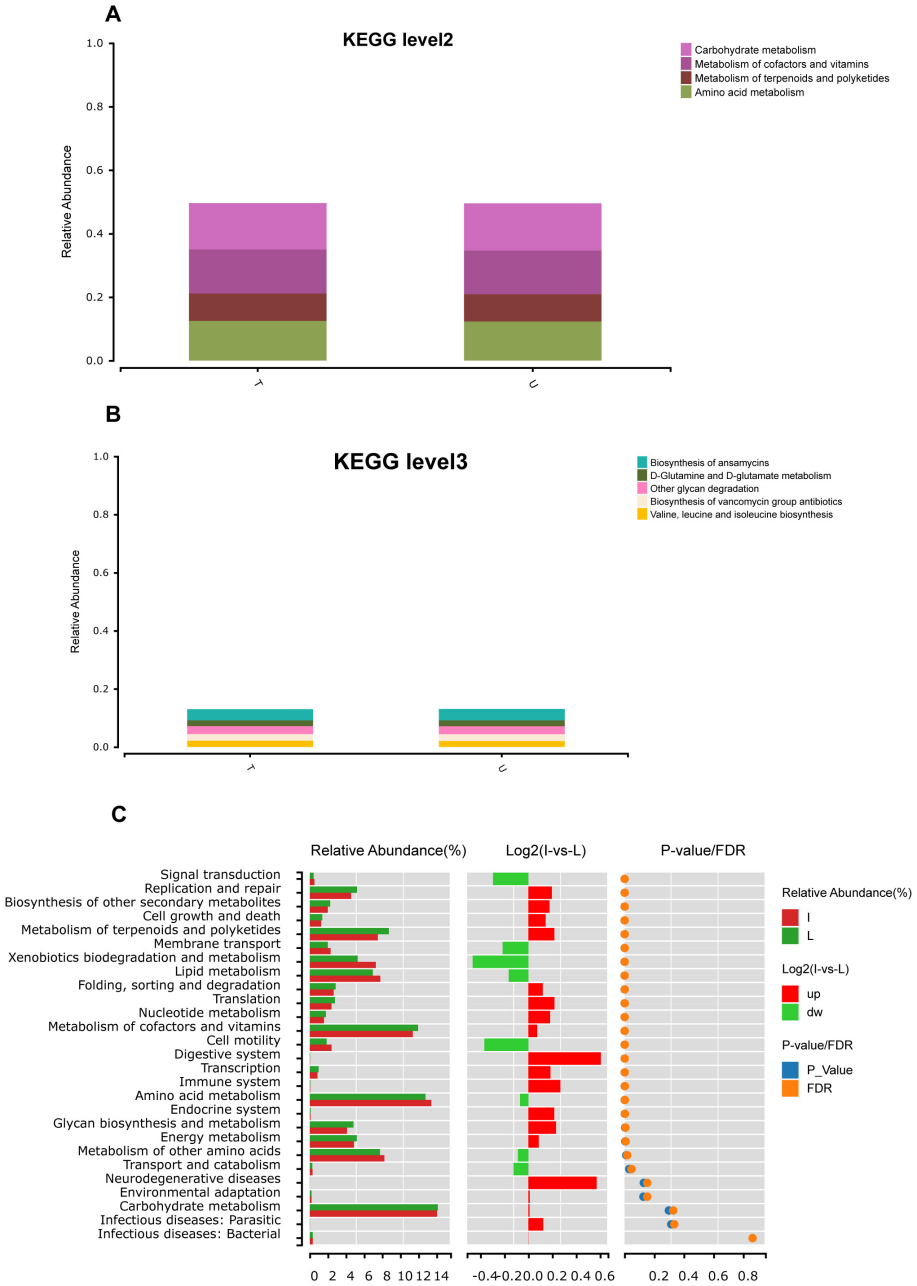
Functional prediction based on PICRUSt2. **(A)** Abundance prediction results of KEGG function of group T and group U bacterial communities at level2 obtained by PICRUST2, abundance threshold is 0.07. **(B)** Abundance prediction results of KEGG function in group T and group U bacterial communities at level3 obtained by PICRUST2, abundance threshold is 0.02. **(C)** After the prediction of the function of all samples was obtained by PICRUST2, the Wilcox test was used to analyze the difference function between group I and group L.

The results of the abundance prediction for Kyoto Encyclopedia of Genes and Genomes(KEGG) functions of bacterial communities were obtained utilizing PICRUST2. The Figure shows that the U and T groups exhibited similar functional profiles at the KEGG level 2 pathway. Enrichment of pathways such as carbohydrate metabolism, metabolism of cofactors and vitamins, amino acid metabolism, and metabolism of terpenoids and polyketides were observed in both groups T and U (**Figure 5A**). At KEGG level 3, pathways such as biosynthesis of ansamycins, other glycan degradation, biosynthesis of vancomycin group antibiotics, valine, leucine, and isoleucine biosynthesis, and D-glutamine and D-glutamate metabolism were enriched in both groups T and U (**Figure 5B**). The microorganisms found in HCC tumors differed significantly from those in normal liver tissues in terms of functional abundance prediction. There were 20 significantly different pathways, mainly enriched in carbohydrate metabolism, amino acid metabolism, and metabolism of cofactors and vitamins (**Figure 5C**).

## 4 Discussion

HCC is a common gastrointestinal tumor, and there is new hope for HCC patients through a triple therapy approach that combines TKIs and PD-1 inhibitors with local therapy. Gut microbiota has emerged as targeted biomarkers for early HCC diagnosis and a non-invasive screening tool. Ren et al. (22) conducted the first comprehensive study of gut microbiota in HCC patients through macro-genome sequencing. They successfully developed a diagnostic model for HCC and validated microbial markers across different regions. Additionally, Lee et al. (23) discovered that gut microbes were linked to the therapeutic response of uHCC to PD-1 inhibitors using 16S rRNA gene sequencing. Gut microbiota is expected to be biomarkers for predicting the impact of PD-1 inhibitors on uHCC treatment. Furthermore, they can potentially be modified to heighten the effect of PD-1 inhibitors in treating HCC by altering the gut microbiota. Prior research has demonstrated a connection between gut microbiota and the clinical response to immunotherapy, suggesting that gut microbiota could serve as a predictive marker for immunotherapy response (24). A notable discrepancy in gut microbiota was identified in a study of HCC patients who exhibited a favorable response to PD-1 inhibitors, as compared to those who did not, and two specific bacteria (*Actinomyces* sp. *ICM47* and *Senegalimassilia anaerobia*), as well as a metabolite (galanthaminone), were ascertained as prospective forecasted biomarkers for subsistence in PD-1 inhibitor-treated HCC patients (25). Our current study analyzed the potential of microorganisms, including gut and intratumoral microbiota, as biomarkers in triple therapy for HCC. We found significant differences in gut microbiota between HCC patients receiving the triple therapy of local therapy with lenvatinib and PD-1 inhibitors and untreated HCC patients. Additionally, we found significant differences in gut microbiota between these two groups when local therapy was either TACE or HAIC. Meanwhile, we verified that the diversity and plenteousness of microorganisms in HCC tumor tissues and normal liver tissues were significantly different and found that the gut microbiota of HCC patients were the same as those of intratumoral microbiota with a high richness of microorganisms at the phylum level, which may explain the origin of some of the intratumoral microbiota.

Lenvatinib was found to have a median survival time non-inferior to sorafenib in a multicenter, noninferiority phase 3 trial (26). With lenvatinib’s increasing use in clinical practice (27), a study was conducted to characterize the gut microbiota and biomarkers of HCC treated with local therapy combined with lenvatinib and PD-1 inhibitors. The study included 26 patients with previously untreated HCC and 26 patients with HCC treated with the triple therapy of local therapy combined with lenvatinib and PD-1 inhibitors and analyzed the microbiological communities by 16S rRNA gene amplicon sequencing. In our investigation, we discerned an augmentation in the α-diversity of gut microbes in group T compared to group U, which refers to the number of species and their relative plenteousness within a sample. However, there was no meaningful variation in the amounts of species and their relative abundance within the gut microbiota of the two groups. We also found that β-diversity, which reflects whether there is a notable divergence in microbial communities between the sample groups, was significantly different between the two groups. Fecal microbes from triple-treated HCC patients had higher species and abundance than those from untreated HCC patients and were significantly variable, suggesting the potential for gut microbes to serve as biological markers of triple-treated HCC. We observed differences between two groups of bacteria, Actinobacteriota and *Escherichia Shigella*, at the phylum and genus levels. There was a remarkable enhancement in the richness of Actinobacteriota and a remarkable reduction in the richness of *Escherichia Shigella* in the T group. Actinobacteriota can generate greater numbers of short-chain fatty acids, which helps to maintain intestinal barrier homeostasis. Butyrate, a type of short-chain fatty acid, can enhance the anti-inflammatory effects of Kupffer cells in the liver (28). Actinobacteriota, particularly *Bifidobacteria*, can regulate immunoinflammatory and autoimmune responses by inducing regulatory T cells (29). On the other hand, increased *Escherichia Shigella* abundance is associated with peripheral inflammatory status, and the richness of *Escherichia Shigella* actively correlates with levels of inflammatory mediators (30). Additionally, a significant correlational relationship was identified between the abundance of *Escherichia Shigella* and the levels of four metabolites, which could potentially influence disease progression (31). Changes in the abundance of Actinobacteriota and *Escherichia Shigella* in HCC patients treated with the triple therapy of local therapy combined with lenvatinib and PD-1 inhibitors may be related to the triple therapy effect. This treatment led to a significant increase in anti-inflammatory microorganisms and a significant decrease in pro-inflammatory microorganisms, enhancing the body’s ability to fight against tumors. Therefore, it is believed that a synergistic triple therapy, involving an increase in Actinobacteriota and a decrease in *Escherichia Shigella*, enhances the efficacy of localized therapy combined with triple therapy using lenvatinib and PD-1 inhibitors. The KEGG pathways in both the T and U groups were similar, primarily enriched in carbohydrate metabolism and amino acid metabolism. In HCC cells, it was observed that the catabolic activation of branched-chain amino acids (valine, leucine, and isoleucine) occurred in the absence of glutamine. Furthermore, dephosphorylation of Branched Chain Keto Acid Dehydrogenase E1 Subunit Alpha (BCKDHA) and high expression of Protein Phosphatase, Mg2+/Mn2+ Dependent 1K (PPM1K) were found to promote tumorigenesis both *in vivo* and *in vitro* and were closely associated with poor prognosis in HCC patients. Inhibition of branched-chain amino acid and glutamine metabolism was also found to retard HCC growth *in vivo* (32). Our sequencing results indicated a higher enrichment of the branched-chain amino acid metabolic pathway in the gut microbiota of both primary HCC patients and patients undergoing triple therapy. At the KEGG level 3, gut microbes were enriched in valine, leucine, and isoleucine biosynthesis, as well as D-glutamine and D-glutamate metabolic pathways, suggesting that gut microbes may achieve antitumor effects through metabolites.

TACE is the standard treatment for patients with intermediate-stage hepatocellular carcinoma and specific advanced hepatocellular carcinoma. The majority of guidelines and consensus statements recommend TACE as the standard first-line treatment option for intermediate-stage HCC (33). HAIC is considered a promising treatment modality for locally advanced HCC. However, due to the lack of sufficient evidence, it is not a standard of care worldwide. Nevertheless, it has been incorporated into the In Asia, particularly in Japan and Korea, guidelines have identified HAIC as an effective treatment option for intermediate-stage HCC. Additionally, guidelines have incorporated HAIC as a viable approach for treating intermediate and advanced hepatocellular carcinoma (34). While TACE and HAIC have demonstrated efficacy as local therapeutic options for hepatocellular carcinoma, the optimal approach remains undetermined. Consequently, our triple therapy incorporates both TACE and HAIC techniques. A multicenter retrospective study demonstrated that FOLFOX-based HAIC in conjunction with TKI and PD-1 inhibitors therapy for HCC markedly enhanced patients’ survival prognosis in comparison to TKI in conjunction with PD-1 inhibitors therapy (35). The majority of current studies concentrate on the relationship between the triple therapy of TACE combined with TKI and PD-1 inhibitors for HCC and gut microbiota. However, it remains unclear which of these approaches is more effective: local therapy combined with TKI and PD-1 inhibitors for HCC or HAIC combined with TKI and PD-1 inhibitors for HCC (36). Subsequently, an in-depth examination of the gut microbiota was conducted on patients who had undergone treatment with distinct triple therapy within Group T. Nine patients received the triple therapy of TACE in combination with lenvatinib and PD-1 inhibitors, while 16 patients received the triple therapy of HAIC in combination with lenvatinib and PD-1 inhibitors. A significant difference was observed in β-diversity between the TACE and HAIC groups. The findings of the lefse analysis suggested that Veillonellales Selenomonadales and *Veillonella* were biomarkers for the TACE group, while Clostridia, *Oscillospirales*, Ruminococcaceae, and *Faecalibacterium* were identified as biomarkers for the HAIC group. Our findings revealed discrepancies at the level of outline and species within the Clostridia, *Faecalibacterium prausnitzii*, and *Bacteroides stercoris* groups. Low levels of *Faecalibacterium* have been associated with a variety of diseases, particularly inflammatory diseases (37). In a study comparing cirrhotic patients with healthy controls, cirrhotic patients exhibited a reduced abundance of gut microbial *Faecalibacterium prausnitzii* strains (38). *Faecalibacterium prausnitzii* has been demonstrated to mitigate PD-1 inhibitor-induced colitis and augment the antitumor efficacy of immunotherapy (39). A recent study presented at the European Lung Cancer Congress 2024 has revealed that the absence of *Faecalibacterium prausnitzii* is connected to improved survival outcomes in patients with locally advanced non-small cell lung cancer treated with perioperative navulizumab and chemotherapy (40). Dietary supplementation with resistant starch may represent a novel strategy for treating nonalcoholic fatty liver disease. Its mechanism of action involves variations in the composition and function of the gut microbiota, with resistant starch inhibiting the key functional bacterium, *Bacteroides stercoris*, to reduce the production of intestinal-derived branched-chain amino acids and enhance hepatic lipid metabolism (41). The abundance of *Faecalibacterium prausnitzii* was greater in the HACI group, indicating that this bacterium may serve as a promising biomarker for predicting the selection of HACI in patients undergoing triple therapy with local therapy in combination with lenvatinib and PD-1 inhibitors. Furthermore, the adjunctive oral administration of triple therapy in HCC patients undergoing *Faecalibacterium prausnitzii* may enhance the efficacy of triple therapy.

Intratumoral microbiota, which may be related to tumor development, have been a focus of research over the past several years. Research has shown that the bacterial diversification in HBV-associated HCC tissues is lower compared to chronic hepatitis tissues. Furthermore, the screening of genetic risk profiles containing CSAG4, PIP4P2, and TOMM5 among dominant bacterial subtypes is capable of being utilized in predicting the clinical prognosis of HCC patients (42). Additionally, the microorganism community in HCC tissues is markedly elevated in comparison to that observed in neighboring tissues, with an increased abundance of microorganisms such as *Enterobacteriaceae*, *Fusobacterium* and *Neisseria*. Certain anti-tumor bacteria, such as *Pseudomonas*, were found to have decreased abundance in HCC tissues (43). We conducted a study comparing the microbial diversity and abundance within liver tumors of HCC patients with that of normal liver tissues. We used 16S rRNA gene amplicon sequencing and identified seven significantly different microbes at the phylum level and 18 significantly varied microorganisms at the genus level. *Burkholderia Caballeronia Paraburkholderia* was most abundant in HCC tissues and normal liver tissues, which had not been found in previous studies of HCC tissues and para-cancerous tissues. Our findings showed that microbial diversity within HCC tumors was significantly lower than in normal liver tissues. Additionally, we discovered a meaningful decrease in the abundance of *Bacteroides*, *Prevotella*, and *Parabacteroides*. HCC tissues exhibit major metabolic alterations in aerobic glycolysis, lipid metabolism, and amino acid metabolism (44). Furthermore, gut microbiota and intratumoral microbiota can influence the TME through metabolites. Additionally, research demonstrated that an antibiotic-induced imbalance of gut flora significantly promoted hepatocarcinogenesis and hepatic cholesterol synthesis, hepatic cholesterol metabolism, induced by gut dysbiosis, was associated with intestinal tryptophan metabolites and the activation of the aromatic hydrocarbon receptor (45). Recent research has shown that butyrate, a metabolite of the intratumoral microbiota *Roseburia*, can facilitate the migration and infestation of lung cancer cells. Additionally, it has been observed that low concentrations of butyrate may contribute to tumor metastasis by upregulating H19-MMP15 and promoting M2 macrophage polarization (46). The present study revealed that microorganisms in HCC tumors were mainly enriched in three pathways: Carbohydrate metabolism, Amino acid metabolism, and Metabolism of cofactors and vitamins. These findings suggest that intratumoral microbiota is engaged in the development and progression of HCC through their involvement in metabolism and metabolites. Tremendous differences in microbial diversity and abundance were discovered between HCC tumor tissues, paracancerous tissues, and normal liver tissues. This demonstrates that intratumoral microbiota has an impact on identifying HCC and participate in its evolution. Identifying and intervening in HCC-targeted intratumoral microbiota may have a beneficial synergistic effect in anti-tumor therapy.

Previous concepts suggested that tumors were sterile, but recent studies confirmed the presence of microorganisms in tumors. The source of these microorganisms in tumors is still unclear and could be due to translocation of gut microbiota, circulatory metastasis, or metastasis to adjacent tissues (47). Microbes from the oral cavity, gastrointestinal tract, and other potential sources may be transported to the tumor site via the bloodstream, subsequently infiltrating the tumor through damaged blood vessels. In addition to the mucus and intestinal epithelial barriers, the gut is equipped with an intestinal vascular barrier, which serves as a gatekeeper, regulating the entry of molecules and microorganisms into the systemic circulation. Following the invasion of the intestinal epithelial cells, the enteric pathogen Salmonella enterica enterica rapidly enters the bloodstream via intestinal CX3CR1 macrophages. The hematogenous pathway represents the primary mode of Salmonella transfer from the gastrointestinal tract to the liver and spleen (48). The bacteria then gain access to the tumor tissue, where they modify the intestinal vascular barrier. They subsequently migrate to the liver, where they promote the formation of a pre-metastatic ecological niche, thus creating a suitable environment for subsequent inoculation of cancer cells. It was demonstrated that the *Escherichia coli* strain (C17) is capable of directly opening the intestinal vascular barrier through a TTSS virulence factor (Virf)-dependent mechanism, subsequently migrating into the liver. There, it initiates immune cell recruitment, which contributes to the maturation of the pre-metastatic ecotope and facilitates metastasis formation (49). The presence of the liver-gut axis allows microbes to transfer between the intestine and the liver, and the gut microbiota may be the source of microbes in HCC. Analysis of the sequencing results of the 16S rRNA gene amplicons of gut microbiota from HCC patients and microbes from the tumors revealed that these two groups of highly abundant microbes were the same at the phylum level. This does not mean that the gut microbiota and the microbiota from the tumors are identical, but there is a certain correlation. The gut microbiota of HCC patients who underwent triple therapy with local therapy combined with lenvatinib and PD-1 inhibitors exhibited structural similarities to those of patients with primary untreated HCC at the phylum level. The most abundant microbiota in these patients was members of the Bacteroidota, Firmicutes, and Proteobacteria. Additionally, the microbiota in HCC tumor tissues was predominantly Proteobacteria, Bacteroidota, and Firmicutes, which mirrored the structure of HCC gut microbiota. The principal altered microbial groups following triple therapy were Actinobacteriota and *Escherichia Shigella*. No such distinction was observed when comparing the microbial profiles of HCC tumors with those of the gut. This suggests a correlation between HCC intratumoral and gut microorganisms, with the observed differences being intrinsic and unrelated to triple therapy. Dysbiosis is a key driver of the development of nonalcoholic steatohepatitis/fatty hepatitis, which can progress to HCC (50). Fecal microbiota transplantation (FMT) has emerged as a promising therapeutic approach for the treatment of dysbiosis-associated diseases (51). The relationship between gut microbiota and intratumoral microbiota with respect to tumor development, anti-tumor therapy, and screening of tumor markers is a topic of great scientific and clinical value, and further exploration of this relationship is warranted.

Our study has several limitations. Primarily, the efficacy of local therapy combined with target immunotherapy as an emerging treatment modality is not guaranteed. Furthermore, this treatment modality has not been universally adopted in all countries. As the study progresses, the advantages of triple therapy will become increasingly evident, and our study will contribute to this understanding. Secondly, the sample size is insufficient and all the patients with successful collection of gut microbiota were male. Although our sample was not specifically screened for male bias, the final enrollment was all male, which may result in errors when calculating the results. To address this, future studies will employ a larger sample size, including multi-center and large-sample studies, to obtain more robust results. Ultimately, the 16S rRNA sequencing technology employed in our study yields limited information. Currently, the advancement of sequencing technology has led to significant advancements in the study of gut microbiota, with the introduction of macro-genome sequencing. Similarly, for intratumoral microbiota, the use of immunohistochemistry and fluorescence *in-situ* hybridization (FISH) technology will markedly enhance the detection rate and accuracy.In subsequent studies, we will employ these techniques further to elucidate the relationship between microorganisms and HCC.

In conclusion, gut microbiota could serve as a potential biomarker for triple therapy when local therapy is combined with lenvatinib and PD-1 inhibitors for HCC. Furthermore, it is possible to anticipate the specific local therapy to be employed, such as TACE or HAIC. Additionally, investigating the interrelationship between gut and intratumoral microbiota may facilitate the elucidation of their interconnection. This understanding has the potential to promote the development of targeted microbial adjuvant therapy for HCC with a broad range of potential applications.

## Data Availability

The datasets presented in this study can be found in online repositories. The names of the repository/repositories and accession number(s) can be found below: https://www.ncbi.nlm.nih.gov/, accession number PRJNA1162776 (release date 01-06-2025).
